# Endoscopic management of biliary fascioliasis: a case report

**DOI:** 10.1186/1752-1947-4-83

**Published:** 2010-03-06

**Authors:** Rajan F Ezzat, Taha A Karboli, Kalandar A Kasnazani, Adnan MH Hamawandi

**Affiliations:** 1 Kurdistan Gastrointestinal Center, Sulaimanyah Teaching Hospital, Sulaimanyah, Iraq

## Abstract

**Introduction:**

*Fasciola hepatica*, an endemic parasite common in Iraq and its neighboring countries, is a very rare cause of cholestasis worldwide. Humans can become definitive hosts of this parasite through their ingestion of a contaminated water plant, for example, contaminated watercress. Symptoms of cholestasis may appear suddenly and, in some cases, are preceded by long periods of fever, eosinophilia, and vague gastrointestinal symptoms. Here we report the case of a woman with a sudden onset of symptoms of cholangitis. Her infection was proved by endoscopic retrograde cholangiography to be due to *Fasciola hepatica *infestation.

**Case presentation:**

A 38-year-old Kurdish woman from the northern region of Iraq presented with fever, right upper quadrant abdominal pain, and jaundice. An examination of the patient revealed elevated total serum bilirubin and liver enzymes. An ultrasonography also showed a dilatation of her common bile duct. During endoscopic retrograde cholangiopancreatography, a filling defect was identified in her common bile duct. After sphincterotomy and balloon extraction, one live *Fasiola hepatica *was extracted and physically removed.

**Conclusion:**

*Fasciola hepatica *should be a part of the differential diagnosis of common bile duct obstruction. When endoscopic retrograde cholangiopancreatography is available, the disease can be easily diagnosed and treated.

## Introduction

*Fasciola hepatica *(FH) is a leaf-shaped trematode that usually attacks cattle and sheep. Humans can become accidental hosts through drinking contaminated water or ingesting raw green vegetables contaminated with encysted metacercariae. The bacteria's larva penetrates the intestinal wall to enter the peritoneal cavity. It then usually passes through the liver capsule and hepatic tissues where, after it becomes an adult, finally invades the biliary tract [1]. The greatest number of infected people has been reported in Bolivia, China, Ecuador, Egypt, France, Iran, Peru, and Portugal. In Iraq, Lebanon, Morocco, Tunisia and Yemen fewer than 100 cases have been documented so far, implying that the problem has probably not yet received enough attention in these countries.

Infestation with FH has two distinct clinical phases: one corresponding to the hepatic migratory phase of the life cycle of the flukes, and the other corresponding to the presence of the parasites in their final location in the bile ducts. FH infestation may be suspected in patients who exhibit tender hepatomegaly, fever, and eosonophilia. Adult flukes can cause obstructive jaundice or make a patient vulnerable to cholelithiasis [2]. Here we report a case of FH infestation that was diagnosed endoscopically and treated by endoscopic extraction along with antiparasitic medication.

## Case presentation

A 38-year-old Kurdish woman from the northern region of Iraq presented with fever, right upper quadrant pain, and jaundice for three days. She had two cesarean sections in 1995 and 2003 and an appendicectomy three years prior to presentation. Six months prior to presentation, she underwent laproscopic cholecystectomy, in which no parasites were found in her gall bladder.

Her physical examination revealed jaundice, scars of previous surgical procedures, and right subcostal tenderness without hepatomegaly. Her laboratory investigations revealed the following results: hematocrit, 30%; white blood cell count, 6700/cmm;eosonophil, 15%: platelets, 169000/cmm; erythrocyte sedimentation rate (ESR), 45 mm/1st hour; alanine aminotransferase, 74 IU/L; aspartate aminotransferase 93 IU/L; gamma glutamyl transferase, 319 IU/L; alkaline phosphatase, 266 IU/L; and total serum bilirubin, 2.2 mg/dl. Results of her ultrasonography revealed normal liver parenchyma, removed gall bladder, normal intrahepatic bile ducts and dilated common bile duct (12 mm). Her stool examination tested negative for ova.

**Figure 1:**
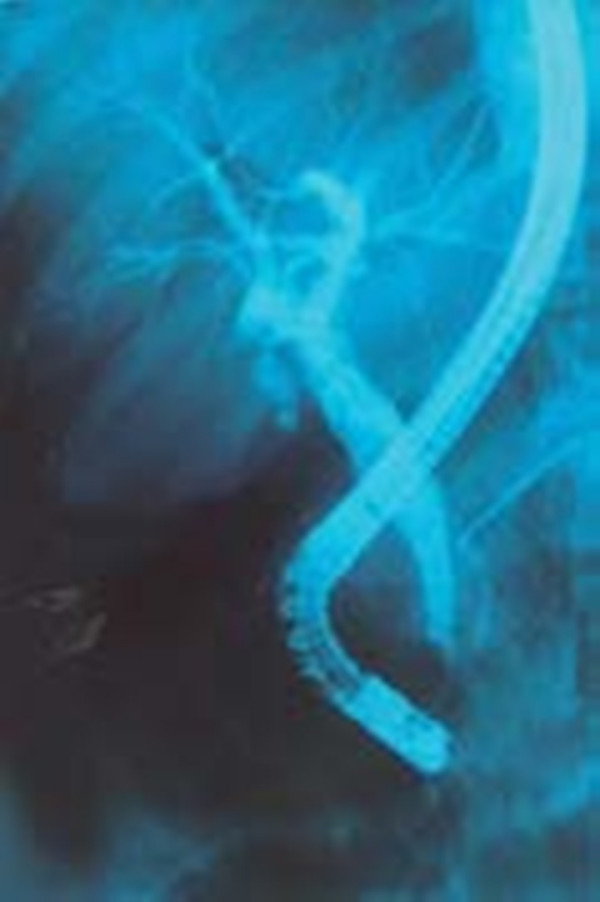
**Cholangiogram revealing a dilated common bile duct**.

**Figure 2:**
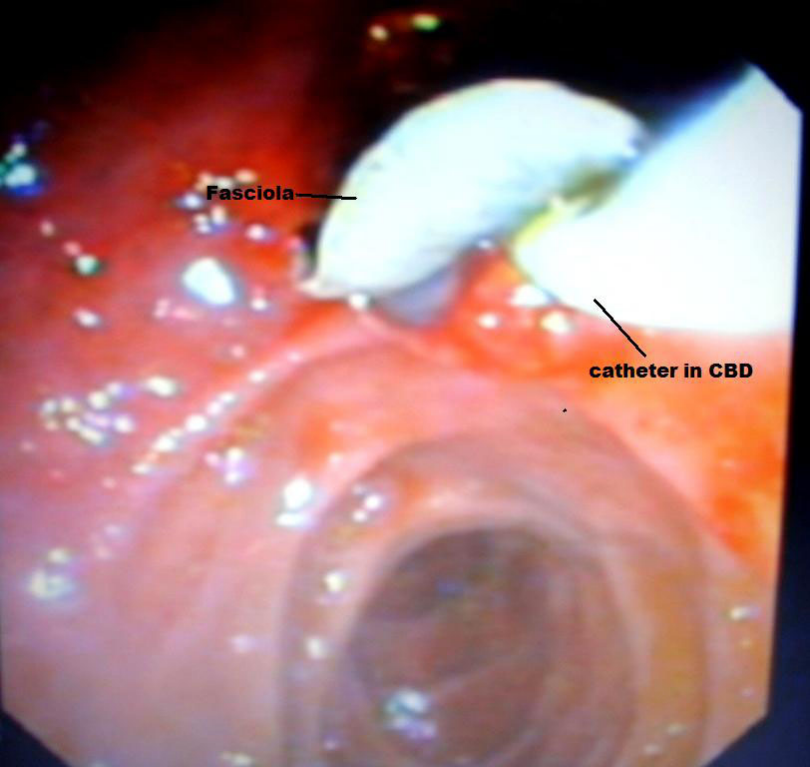
**Fasciola coming out from the choledocus**.

**Figure 3:**
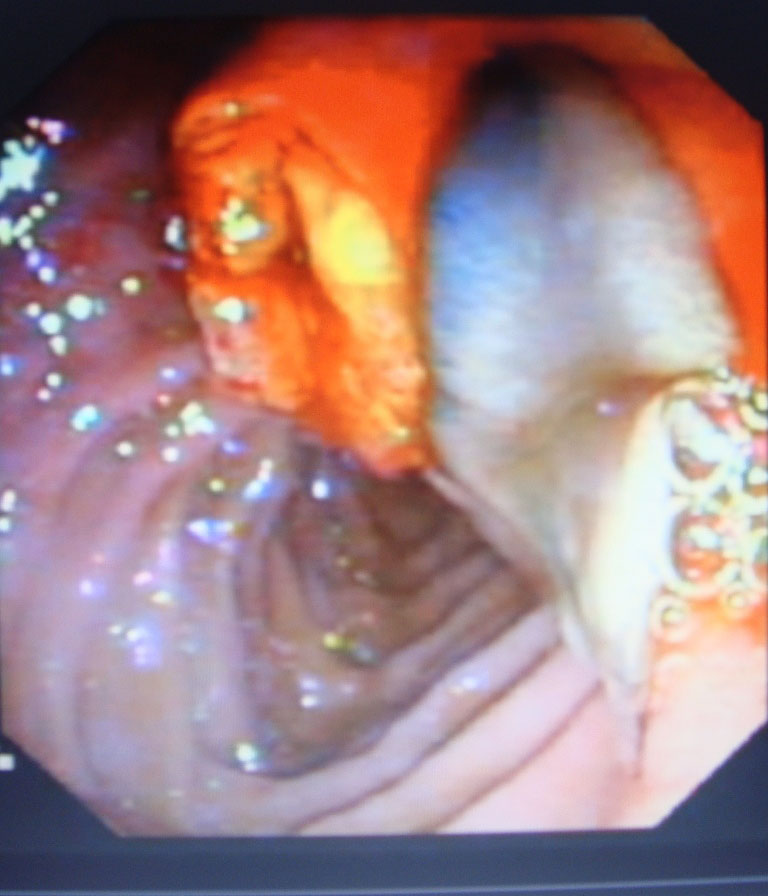
**Fasciola in the duodenum**.

**Figure 4:**
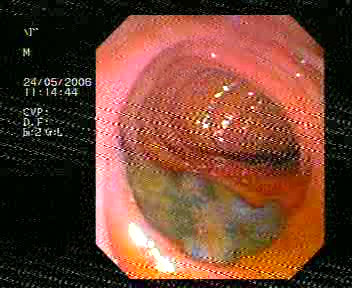
**Fasciola swimming in the duodenum**.

**Figure 5:**
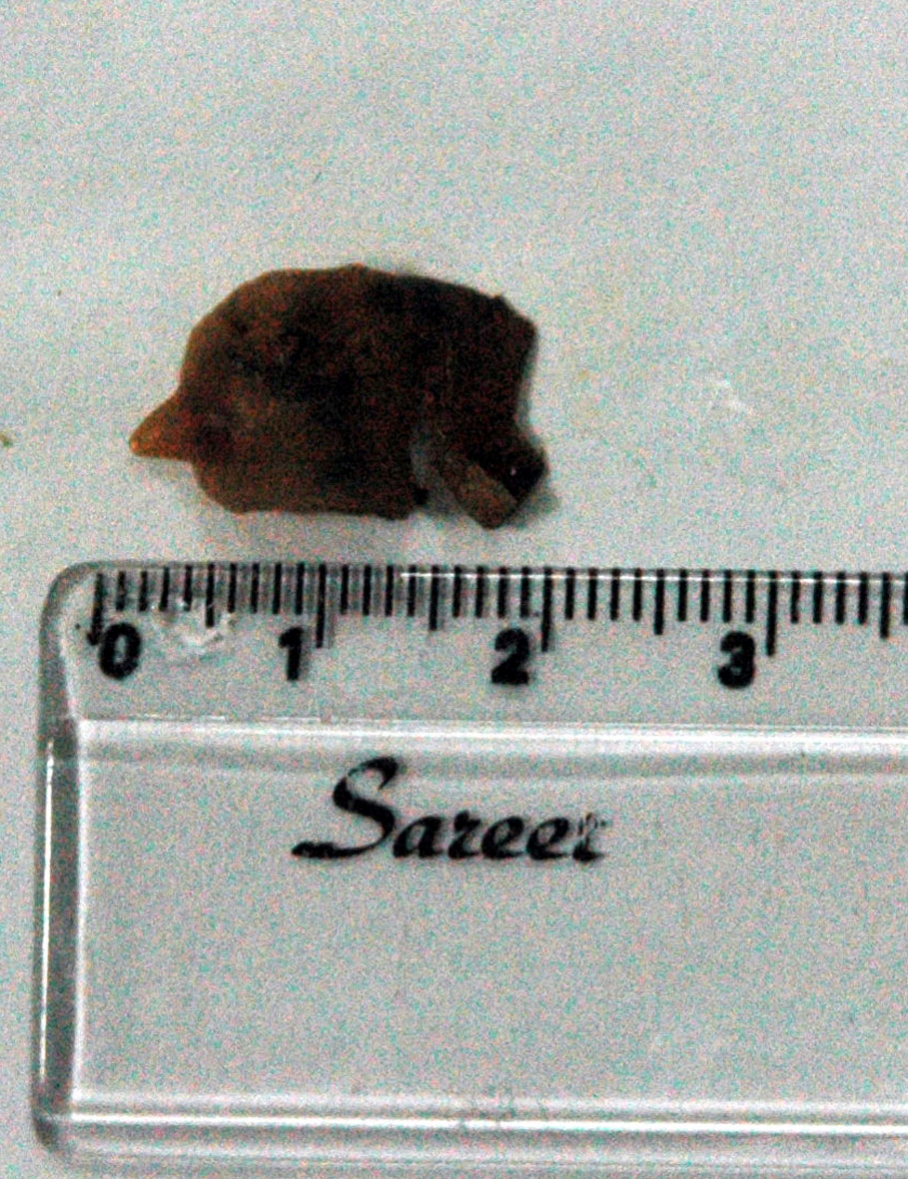
**Fasciola hepatica *in vitro***.

Endoscopic retrograde cholangiopancreatography (ERCP) was done to test for extrahepatic cholestasis. This revealed dilated common bile duct (with a diameter of 11 mm) and a filling defect in her common bile duct (Figure 1). During balloon extraction after her endoscopic sphincterotomy, one live FH was forced through her choledocus (Figure 2) and into the lumen of her duodenum (Figures 3 and 4) and then physically removed by biopsy forceps (Figure 5).

We prescribed our patient with a single morning dose of 10 mg/kg of Tricalbendazole [3]. On her follow-up examination, after four weeks, her liver enzymes had returned to normal. Another ultrasonography revealed that the size of her bile duct was normal.

## Discussion

A variety of liver flukes, including *Fasciola hepatica*, may colonize the biliary tree of humans where they lay their eggs. These eggs can give rise to the formation of gallstones by serving as nidus for them. Living or dead worms may occlude the bile ducts, thus causing obstruction and sometimes cholangitis. Fascioliasis is primarily a common disease of livestock animals such as cattle and sheep, with humans serving occasionally as accidental hosts.

Two stages have been described in human fascioliasis: an acute phase, which coincides with hepatic invasion, and a chronic phase, which develops due to the presence of flukes in the bile ducts [4]. The metacercariae for these parasites encyst on freshwater plants, such as wild watercress. Human consumption of aquatic plants harvested from contaminated areas can lead to infection. Subsequently, the developing larvae penetrate the gut wall and enter the peritoneal cavity. After a period of migration for six to nine weeks, the flukes penetrate the capsule of the liver and mature in the biliary tree and begin to pass their eggs. In the acute phase of the disease, our patient may have prolonged fever, right upper quadrant pain, liver enlargement and eosinophilia that can be easily misdiagnosed. These symptoms abate when the chronic phase is reached. Once the flukes enter the bile ducts, they may cause symptoms due to cholestasis and cholangitis.

Although the definitive diagnosis for fascioliasis can be made by detecting the eggs of the parasite in the stool or duodenal aspirates, egg detection rate is not high because of the low egg production rate of the parasite. Immunoserological tests thus become the basis for the diagnosis of fascioliasis, especially during its early stages or in ectopic infections, but an enzyme-linked immunosorbent assay (ELISA) test provides more rapid and reliable results. Although some parts of Iraq are endemic areas for human fascioliasis, we did not immediately arrive at this diagnosis in the case of our patient. We did not come up with a diagnosis before the parasite reached a full term (ERCP) because *Fasciola hepatica *is still considered as a very rare cause of biliary obstruction. As expected, sphincterotomy and balloon extraction rapidly alleviated our patient's symptoms.

Unlike patients with other liver flukes, therapeutic failure is common in patients with *Fasciola hepatica *treated with praziquantel. Bithionol or triclabendazole remains the treatment of choice for this parasitic infection. The use of bithionol, with a recommended dose of 30 to 50 mg/kg every other day for 10 to 15 doses or repeated doses has resulted in the cure of acute and prolonged fascioliasis. Triclabendazole, another effective and safe drug for fascioliasis, has been found to eradicate the parasite with a single oral dose of 10 mg/kg [4]. Our patient was treated with a single 10 mg/kg oral dose of triclabendazole.

We report this case because fascioliasis should be kept in mind in the treatment of patients with cholestasis and preceding vague gastrointestinal symptoms, especially in endemic areas of the world.

## Conclusion

Chronic biliary fascioliasis may be asymptomatic or it may present with biliary obstruction, cholangitis, or portal fibrosis [5]. Our patient presented with cholangitis, and FH was not suggested during the investigations prior to ERCP when her condition was being diagnosed and treated. However, serological tests can help in arriving at the correct diagnosis [2], although such tests are not available in Kurdistan.

The technique of endoscopic sphincterotomy was initially introduced to treat common bile duct stones. The indications have been expanded to include other biliary disorders. Currently, this method is considered as the optimal approach in treating biliary parasitosis including biliary fascioliasis, biliary ascariasis, and biliary hydatid disease [6-8]. Previous reports have noted success with the combination of ERCP and sphincterotomy for extracting FH from the biliary tree [6,9,10]. This case report emphasizes the inclusion of FH in the differential diagnosis for symptoms of right upper quadrant pain and common bile duct dilatation, particularly when ERCP management is amenable.

## Consent

Written informed consent was obtained from our patient for publication of this case report and any accompanying images. A copy of the written consent is available for review by the Editor-in-Chief of this journal.

## Competing interests

The authors declare that they have no competing interests.

## Authors' contributions

RE collected, analyzed and interpreted our patient's data, and assisted in the therapeutic Endoscopy of our patient. TK performed the endoscopy and assisted in interpretating our patient's data. KK assisted in the therapeutic endoscopy and analyzed our patient's data. AH assisted in analyzing and collecting the patients' data. All authors read and approved the final manuscript.

## References

[B1] Adel AFM Mandell GL, Bennet JE, Dolin R Trematodes and other flukes Principles and Practice of Infectious Diseases 2000 5 Philadelphia: Churchill Livingstone 2954 2956

[B2] Marsden PD Shiff Parasitic disease of the liver Diseases of the Liver 1999 Philadelphia: Lippincott William and Wilkins 1078 1088

[B3] Barrett-Conner E Braude AI Fluke infections Infectious Diseases and Medical Microbiology 1986 2 Philadelphia: WB Saunders 979 982

[B4] Lopez VR Dominguez CA Garron C Successful treatment of human fascioliasis with tricalbendazole Eur J Clin Microbiol Infect Dis 1999 18 7 525 526 10.1007/s100960050338 10482035

[B5] Dias LM Silva R Viana HL Palhinhas M Viana RL Biliary fascioliasis: diagnosis, treatment and follow-up by ERCP Gastrointest Endosc 1996 43 616 620 10.1016/S0016-5107(96)70203-1 8781945

[B6] Veerappan A Siegel JH Podany J Prudente R Fasciola hepatica pancreatitis: endoscopic extraction of live parasite Gastroinest Endosc 1991 37 437 435 10.1016/s0016-5107(91)70784-0 1916172

[B7] Khuroo MS Zargar SA Mahajan R Hepatobiliary and pancreatic ascariasis In India Lancet 1990 335 1503 1506 10.1016/0140-6736(90)93037-P 1972440

[B8] Alkarawi MA Yasawy MI Mohamed A Endoscopic management of biliary hydatid disease: report of six cases Endoscopy 1991 23 278 281 10.1055/s-2007-1010686 1743129

[B9] Ozer B Serin E Gümürdülü Y Gür G Yilmaz U Boyacioğlu S Endoscopic extraction of living fasciola hepatica: case report and review of literature Turk J Gastroenterol 2003 14 1 74 77 14593544

[B10] Bafandeh Y Daghestani D Rad S Biliary tract obstruction due to fasciola hepatica managed by ERCP IJMS 2003 28 1 43 45

